# Authentication of Propolis: Integrating Chemical Profiling, Data Analysis and International Standardization—A Review

**DOI:** 10.3390/foods14244259

**Published:** 2025-12-10

**Authors:** Kristian Pastor, Slobodan Dolašević, Nataša Nastić

**Affiliations:** 1 Faculty of Technology Novi Sad, University of Novi Sad, Bulevar cara Lazara 1, 21000 Novi Sad, Serbia; natasa.nastic@uns.ac.rs; 2 Institute of Animal Husbandry, Autoput Beograd-Zagreb 16, 11000 Belgrade, Serbia; dolasevicslobodan.izs@gmail.com

**Keywords:** bee products, chemical fingerprinting, biomarkers, chromatography, spectroscopy, metabolomics, multivariate statistics, machine learning, quality control

## Abstract

Propolis is an apicultural product known for its antioxidant, antimicrobial and anti-inflammatory properties. However, its composition varies with botanical sources, geography, season and bee species, complicating quality control and creating opportunities for adulteration, such as the addition of poplar bud extracts or non-propolis resins. This review synthesizes the latest primary studies and reviews addressing chemical markers identified through analytical platforms, such as TLC, HPTLC, HPLC, LC-MS, GC-MS, NMR, FTIR and ICP, often integrated with chemometrics and machine learning for authentication and standardization. Marker panels are linked to regional chemotypes, including poplar-type, Brazilian green, red and brown, Cuban variants, and stingless bee propolis. Fraud detection strategies using marker-based screening and spectral pattern recognition are also summarized. Multi-marker and chemometric approaches consistently differentiate botanical types, origins and commercial extracts. Common marker families include flavonoids (pinocembrin, chrysin, galangin), phenolic esters (CAPE, benzyl/allyl caffeates), prenylated cinnamates like artepillin C, lignans, and volatile terpenoids or benzenoids. Rapid screening by ATR-FTIR and NMR is often complemented with LC-MS for confirmatory quantitation. Propolis quality control is moving toward harmonized workflows combining FTIR/NMR/HPTLC screening with LC-MS verification and optional elemental or volatile profiling, paving the way for shared marker sets and international standards similar to those for honey.

## 1. Introduction

As a natural bee product, propolis represents one of the most remarkable examples of insect–plant interactions [1]. Through evolutionary adaptation honeybees developed the ability to collect plant resins and transform them into a multifunctional material used for sealing, protection and defense within the hive [2]. Bees gather sticky exudates from buds, bark or other plant parts and mix them with wax and salivary enzymes, producing a resinous substance of complex chemical nature [3]. Within the colony, propolis acts both as a physical barrier and an antimicrobial agent, preventing microbial growth and reinforcing structural stability [4]. Some solitary and stingless bees also use similar plant resins to line their nests or narrow the entrance, showing that this protective behavior is common among different bee species, not only honeybees [5].

Chemically, propolis consists of approximately 50% plant resins and balsams, 305 beeswax, 10% essential oils, 5% pollen and other organic constituents, including aldehydes, alcohols, fatty acids, aliphatic hydrocarbons, sugar derivatives, phenolics, proteins and amino acids [6]. Although it has long been reported that more than 300 different chemical constituents have been identified in propolis samples, including flavonoids, phenolic acids, terpenes, aromatic aldehydes and esters, with their relative abundance varying widely among samples [7,8], more recent studies indicate that this number is considerably higher. Over 800 compounds have been reported in different types of propolis, including temperate, tropical, birch, Mediterranean, and Pacific propolis; these mainly include alcohols, acids and their esters, benzofuranes, benzopyranes, chalcones, flavonoids and their esters, glycosides (flavonoid and diterpene), glycerol and its esters, lignans, phenylpropanoids, steroids, terpenes, and terpenoids [9]. The chemical variability of propolis is primarily influenced by the botanical origin of the resins, environmental factors and bee species [10,11].

From an economic and health perspective, propolis holds a significant position among bee products, together with honey, pollen and royal jelly [12]. In addition to widely used plant-based resources known for their diverse biological activities [13,14], propolis stands out for its broad spectrum of bioactive properties, including antibacterial, antiviral, antioxidant, and anti-inflammatory effects. These characteristics have led to its extensive application in the pharmaceutical, cosmetic, and food industries [2,15]. During the COVID-19 pandemic, its antiviral activity against SARS-CoV-2 further highlighted its pharmacological potential [16]. Recent studies also highlight that propolis and other bee products possess a therapeutic potential also in veterinary medicine, where in vivo studies have demonstrated healing, anti-inflammatory and immunomodulatory effects in multiple animal species [17]. Propolis is commercially available as ethanolic or aqueous tinctures, capsules, powders and extracts [18]. However, despite its global market expansion, no unified international quality standards or characterization parameters have yet been established [19,20].

The aim of this review is to synthesize current advances in propolis authentication by integrating analytical techniques, chemometric and machine learning tools and emerging standardization efforts. We summarize diagnostic marker compounds across global chemotypes, describe analytical workflows for botanical and geographical discrimination, and highlight how multivariate and machine-learning tools enable reproducible classification and support the development of international quality standards.

## 2. Botanical and Geographical Variability

**Figure 1:**
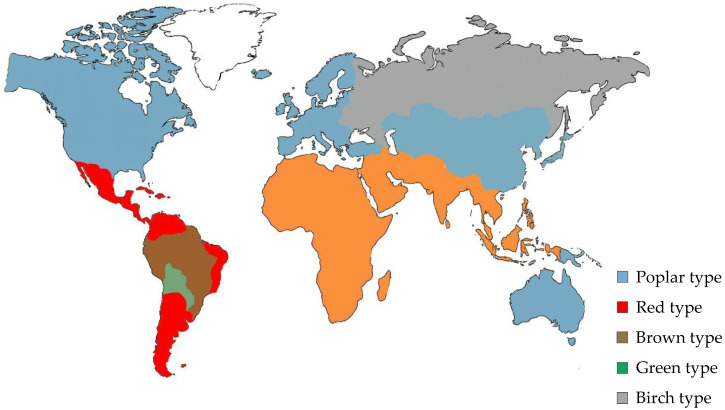
Global distribution of major propolis chemotypes.

The botanical origin of propolis largely determines its chemical composition. Based on the dominant plant resins collected by bees, seven major chemotypes have been described in the literature: poplar, birch, green, red, brown and mixed-type (Figure 1) [21,22,23,24,25]. These categories reflect predominant resin-based chemotypes rather than an exhaustive global classification. However, as highlighted by Salatino and Salatino [26], several widely quoted statements regarding the “typical” gross composition of propolis, namely that it contains “50% resins and balsams, 30% wax, 5% pollen and 10% essential oils” [21,22,23,24,25], are generic, unsupported by analytical methodology and should not be interpreted as universal descriptors. Their analysis shows that these values have been reproduced uncritically for decades despite significant geographical, botanical and ecological variability. In fact, when robust analytical methods are applied, propolis may exhibit resin contents exceeding 70% or wax contents ranging from <10% to over 40%, depending on local plant sources and ecosystem characteristics. Thus, gross composition values should be interpreted with caution and always contextualized within the specific botanical and geographical origin of the samples examined.

The poplar-type propolis is predominant in Europe, North America and parts of Asia. It is derived mainly from *Populus* species (*P. nigra*, *P. alba*, *P. tremula*), with secondary plant sources such as *Betula pendula*, *Salix alba*, *Pinus* sp. and *Aesculus hippocastanum* [27]. It is characterized by high contents of flavonoid aglycones and esters of substituted cinnamic acids, together with aromatic sesquiterpenes in its volatile fraction [28].

In contrast, Baccharis-type propolis from South America, especially Brazilian green propolis derived from *Baccharis dracunculifolia*, is rich in cinnamic acid derivatives, with artepillin C as a specific marker compound [29]. Red propolis, collected from *Dalbergia ecastophyllum* in northeastern Brazil and Cuba, shows high levels of isoflavonoids, while brown and Mediterranean propolis types exhibit diverse terpenoid and phenolic profiles depending on the vegetation [4,30]. Araucaria-type propolis, found in the southern regions of Brazil, originates from *Araucaria angustifolia* and displays distinct diterpenoid composition.

Geographical conditions, seasonal variation and bee species further influence the resin sources and hence the final chemical profile [31]. Even within a small area, differences in flora or collection time can lead to distinct chromatographic fingerprints, as observed between hives separated by only one kilometer [28]. Stingless bees within the tribes *Meliponini* and *Apini* also produce a variant called geopropolis, by mixing plant resins with soil particles or plant debris, yielding a material of distinct composition and texture [31].

## 3. Fraud and Adulteration in Propolis

The rising demand and limited natural supply of propolis have increased the risk of fraudulent practices and adulteration in commercial products [32,33]. The most common form of adulteration involves the addition of plant extracts, particularly poplar bud extract, since its flavonoid composition closely resembles that of poplar-type propolis [7]. Analytical differentiation can be achieved through detection of catechol, a marker compound of poplar buds that becomes oxidized during enzymatic modification in authentic propolis [7,34].

Another fraudulent approach is dilution with cheaper natural or synthetic resins, such as pine resin or other balsamic materials, which mimic the sticky texture of propolis but lack its bioactive profile. In some cases, manufacturers introduce synthetic flavonoids or phenolic acids to artificially raise total phenolic content and mimic genuine antioxidant activity. Such practices alter chemical fingerprints and can mislead chromatographic or spectrophotometric assessment [7,30,34,35,36].

Other adulteration strategies include excessive dilution with wax, addition of colorants or essential oils, or the use of industrial materials (e.g., asphalt, mineral oil, or paint residues) when bees have limited access to natural resin sources [36]. These impurities not only compromise authenticity but may also introduce toxic compounds, posing potential risks to consumers.

To ensure product integrity, advanced analytical techniques, including high-performance liquid chromatography (HPLC), gas chromatography (GC), Fourier-transform infrared spectroscopy (FTIR) and high-performance thin-layer chromatography (HPTLC), coupled with chemometrics, are increasingly applied for authentication and detection of adulterants [30,35,37]. Chemometric tools such as principal component analysis (PCA), linear discriminant analysis (LDA), and machine learning algorithms allow the development of robust classification models, achieving accuracy above 80% for differentiating authentic from adulterated samples [38,39]. As global trade expands, establishing standardized criteria and marker-based quality control systems remains essential for protecting consumers and maintaining the economic and therapeutic value of genuine propolis.

**Figure 2:**
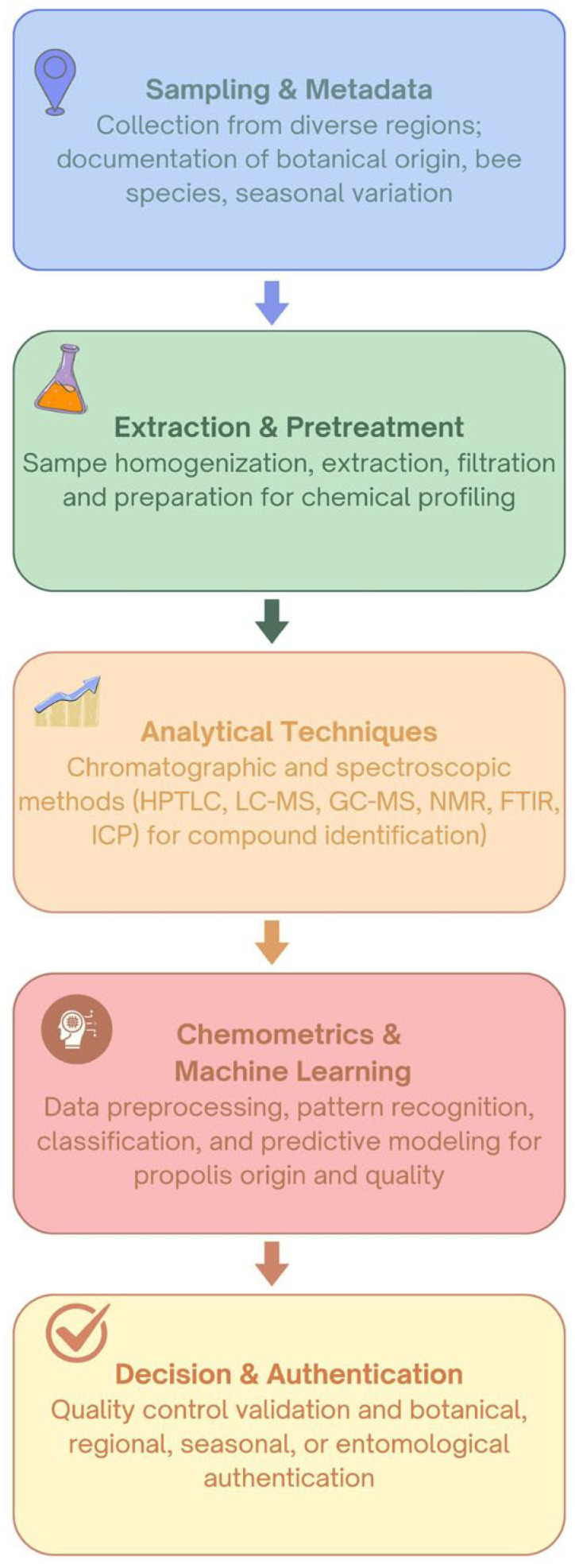
Workflow of propolis authentication and quality control.

Reliable authentication of propolis requires an integrated workflow that connects sampling design, analytical profiling and data interpretation. Each stage, from sample collection and extraction to instrumental analysis and chemometric modeling, contributes to the traceability and reproducibility of results. Figure 2 summarizes this step-by-step framework, illustrating how chromatographic or spectroscopic data are transformed into classification models that validate propolis origin and quality.

The compiled dataset (Table S1) summarizes approximately fifty peer-reviewed studies on propolis authentication from 2005 to 2025, encompassing multiple analytical platforms and geographical regions. Each entry integrates essential metadata including study aim, type and number of propolis samples, bee species, extraction solvent, analytical technique, chemometric or machine-learning method, identified discriminating markers and the key outcomes. The table crosslinks propolis types with their botanical sources, geographical origins and chemical signatures, providing a consolidated reference for understanding global trends in propolis authentication. Summary of some representative studies on propolis (collected by *Apis mellifera* bee) authentication by chromatographic, spectroscopic and other analytical approaches is shown in Table 1.

## 4. Analytical Approaches for Propolis Profiling and Characterization

The authentication of propolis relies on complementary analytical approaches that capture its chemical complexity across different compound classes: phenolics, flavonoids, terpenoids, volatiles, proteins and trace elements. Analytical platforms have evolved from conventional chromatographic quantification toward multidimensional fingerprinting and high-throughput spectroscopic and omics-based methods, often enhanced by chemometric and machine learning analyses. This section summarizes the major analytical techniques applied to discriminate propolis by botanical and geographical origin, classify regional chemotypes and detect adulteration, which are shown in detail in Table S1.

**Figure 3:**
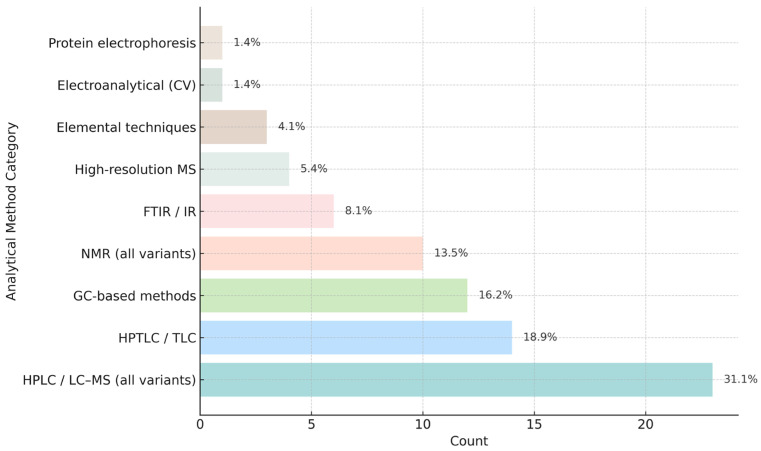
Relative frequency and percentage contribution of instrumental analytical platforms used in 43 propolis authentication studies (2005–2025) from Table S1.

To provide a clearer overview of methodological trends, we quantified the analytical platforms reported across the 63 studies included in Table S1. As shown in Figure 3, chromatographic techniques dominate propolis authentication workflows, with HPLC/LC–MS representing nearly one-third of all methodological mentions. HPTLC/TLC and GC-based approaches collectively account for approximately one-third of all applications, reflecting their importance for phenolic fingerprinting and volatile profiling. Spectroscopic techniques (NMR, FTIR) form the next most common group, while elemental, electroanalytical and proteomic methods remain emerging but complementary approaches.

### 4.1. Chromatographic Techniques

Chromatographic approaches remain fundamental to propolis authentication, enabling both qualitative and quantitative resolution of phenolic acids, flavonoids, terpenoids and other specific marker compounds. Techniques such as HPTLC, HPLC–DAD/MS, UHPLC–HRMS/MS and GC–MS provide complementary insights into botanical origin, chemotype and extraction-dependent compositional profiles, as shown in Figure 3 and Table S1.

Mayworm et al. [37] developed a simple and low-cost TLC method for detecting allyl 3-prenylcinnamate, a volatile marker characteristic of Brazilian green (alecrim) propolis, derived from *Baccharis dracunculifolia*. By heating powdered propolis inside a Petri dish, the sublimed volatiles condensed on the lid and were extracted for TLC analysis using hexane. Plates visualized with sodium fluorescein or sulphuric acid–vanillin reagent showed a clear, well-defined spot at Rf ≈ 0.65, enabling rapid confirmation of the presence of this botanical marker without requiring HPLC or GC instrumentation. The method reliably detected the marker in samples from Minas Gerais and correctly distinguished samples lacking alecrim resin sources.

Güzelmeriç et al. [27] demonstrated that HPTLC phenolic fingerprints combined with image analysis and pattern recognition could successfully classify Turkish propolis into three main chemotypes: orange (*Populus nigra* L.), blue (*Populus tremula* L.) and a newly identified nonphenolic type. Similarly, quantitative HPTLC image analysis was extended using partial least squares (PLS) and genetic inverse least squares (GILS) calibration models to estimate phenolic compound concentrations directly from digitized chromatograms, thereby offering a rapid and environmentally friendly platform for quality control [45].

Morlock et al. [3] introduced a hybrid HPTLC/DART–MS (direct analysis in real time mass spectrometry) workflow, demonstrating that orthogonal datasets significantly improved the differentiation of 91 European propolis samples while reducing analysis time. This platform provided simultaneous separation and soft ionization of phenolic bands, yielding *m*/*z* signatures (e.g., galangin *m*/*z* 269, caffeic acid *m*/*z* 179, chrysin *m*/*z* 253) that strengthened samples’ chemotype assignment.

A comprehensive chromatographic and chemometric investigation of African propolis was provided by Amankwaah et al. [46], who integrated TLC/HPTLC imaging, UHPLC–QTOF–MS, GC–MS and multivariate modeling to characterize Ghanaian propolis extracts. Their results demonstrated that both geographical origin and extraction solvent significantly influenced phenolic profiles, antioxidant capacity and α-amylase inhibition. Ethanol–water extracts showed the strongest bioactivity, with UHPLC–QTOF–MS identifying caffeic-acid derivatives, chlorogenic acid, rosmarinic acid and several flavonoid glycosides (quercetin-4′-O-glucoside, hesperidin, naringenin-C-glucoside). GC–MS further revealed triterpenoids, steroids, lactones, fatty amides and other semi-volatiles unique to African propolis. HPTLC fingerprints clustered samples according to provenance, confirming that African propolis, although chemically distinct, shares polyphenolic features consistent with poplar-type signatures. Bozkuş et al. [19] conducted a comprehensive side-by-side comparison of water and ethanolic extracts of Turkish propolis using HPLC-DAD and GC–MS. HPLC-DAD revealed that hydrophilic phenolic acids dominated the water extract, most notably caffeic acid, chlorogenic acid, and 3,4,5-tri-O-caffeoylquinic acid, while the ethanolic extract was exceptionally rich in lipophilic flavonoids such as chrysin, CAPE, pinocembrin, galangin and naringenin, together with kaempferol, myricetin, quercetin, and trans-cinnamic acid. GC–MS analyses using dual columns (Rtx-1 and Rtx-5ms) complemented these findings by detecting quinic and ferulic acids, cinnamic acid derivatives, benzoic acid and a wide array of sugar derivatives enriched after derivatization, with caffeic acid appearing prominently in ethanolic extracts.

Ristivojević et al. [36] applied UHPLC–LTQ/Orbitrap/MS/MS to characterize Turkish propolis and identified 51 phenolic compounds, confirming its classification as European poplar-type propolis. The authors reported a consistent dominance of flavanones and flavonols (pinocembrin, chrysin, galangin), along with phenolic acid esters (CAPE, benzyl caffeate) that served as key discriminants across regions. Sharp, well-resolved chromatographic peaks and reproducible MS/MS fragmentation signatures enabled differentiation among Turkish provinces, while PCA based on the UHPLC–HRMS dataset successfully separated samples by geographical origin. Sha et al. [47] developed and validated an HPLC-UV method for the simultaneous quantification of eight major phenolic markers, including caffeic acid, p-coumaric acid, ferulic acid, CAPE, pinocembrin, chrysin and galangin, in 19 Chinese propolis samples. Their method showed excellent linearity, precision, and recovery, demonstrating that these compounds form a stable core phenolic profile in Asian poplar-type propolis. Chromatographic abundances varied significantly between regions, reflecting ecological differences and resin-source variability. Cuesta-Rubio et al. [48] combined HPLC-PDA and HPLC–ESI/MS to classify Cuban propolis into three distinct chemotypes: brown, red, and yellow. Chromatographic elution patterns revealed that brown propolis was dominated by polyisoprenylated benzophenones (e.g., nemorosone), red propolis by isoflavonoids and related phenolics, and yellow propolis by aliphatic diterpenoids and other low-UV-absorbing compounds. The chemotype-specific retention time and UV/MS spectral profiles provided clear structural signatures, enabling robust differentiation of tropical non-poplar propolis. Jiang et al. [49] characterized propolis from the Changbai Mountains using HPLC-UV and HPLC–ESI/MS, demonstrating that this material represents a distinct poplar-type chemotype within China. All 21 samples shared highly similar chromatograms enriched in p-coumaric acid, caffeic acid, ferulic acid, and several characteristic flavonoids (pinobanksin, pinocembrin, chrysin, galangin), though with notably elevated levels of p-coumaric acid compared to common Chinese propolis. The authors isolated an unknown late-eluting marker peak, designated CBE (t_n_ = 92 min), and identified it as benzyl p-coumarate, which—together with p-coumaric acid—served as a diagnostic fingerprint for propolis from this region.

Avula et al. [50] applied a comprehensive UHPLC–DAD–QTOF–MS workflow to profile Indian propolis across multiple regions, identifying a wide panel of phenolic acids, flavonoids and benzophenone derivatives typical of the poplar-type chemotype. Their chromatographic fingerprints revealed extensive qualitative variability between samples, with clear differences in the abundance of pinobanksin derivatives, flavonol aglycones and caffeic acid esters, supporting geographical clustering through chemometric analysis. A related study by Pant et al. [51] combined LC-ESI-QTOF-MS with HPLC quantification to characterize 67 phytochemicals in northern Indian propolis, highlighting regional marker compounds, such as proanthocyanidins, isoflavonoids and carotenoids.

Silici and Kutluca [52] used GC–MS to characterize propolis collected by three *Apis mellifera* races in the same apiary, identifying 48 compounds, including many reported for the first time in propolis. Despite being produced in the same environment, the samples showed subtle differences in chromatographic profiles, particularly in flavonoids (naringenin, acacetin), aromatic acids (benzoic, ferulic, cinnamic derivatives), sesquiterpenes (eudesmol, bisabolol) and phenolic aldehydes (vanillin). The predominance of benzoic acid, 3,4-dimethoxycinnamic acid, benzyl benzoate and eudesmol in all samples indicated a shared botanical origin from *Populus alba*, *P. tremuloides* and *Salix alba*. The GC–MS fingerprints demonstrated that even when bees of different races forage in the same location, the resulting propolis remains chemically consistent with regional *Populus/Salix* resin sources.

### 4.2. Spectroscopic Techniques

Spectroscopic techniques, such as FTIR, ultraviolet–visible (UV–Vis) and NMR spectroscopy are rapid, non-destructive alternatives for propolis characterization and screening. FTIR and UV–Vis allow high-throughput screening and pattern recognition, while NMR provides deep structural insight into metabolites, as shown in Table S1.

Badiazaman et al. [53] combined HPTLC and FTIR fingerprinting with PCA/HCA to discriminate Malaysian stingless bee (*G. thoracica*) propolis according to locality. HPTLC revealed differences in phenolics, flavonoids and terpenoids, while FTIR showed clear locality-dependent shifts in several functional-group regions. In particular, broad O–H stretching bands around 3360–3350 cm^−1^ reflected variations in phenolic and alcoholic groups, whereas differences in the CH_2_/CH_3_ stretching region (2931–2914 and 2875–2846 cm^−1^) indicated changes in wax-derived lipids and terpenoid abundance across sampling sites. More discriminating bands were found in the carbonyl region: a unique saturated ketone vibration at 1715 cm^−1^ occurred only in Besut region samples, while conjugated acidic carbonyls (1699–1683 cm^−1^) varied between other localities, reflecting differences in caffeic- and ferulic-acid derivatives. Additional separation arose from aromatic C=C stretching (1597–1587 cm^−1^) linked to flavonoid content and from several C–O/O–H deformation bands in the 1288–1020 cm^−1^ range, associated with primary, secondary and tertiary alcohols and phenolic ester groups. Low-frequency alkene/aromatic bending signals around 887–875 cm^−1^ further distinguished specific regions (Besut vs. Lundang regions).

Surek et al. [54] further demonstrated the discriminative power of ATR–FTIR for classifying Brazilian propolis from *Apis mellifera* (green propolis) and three stingless bee species (tubuna, mandaçaia, and plebeia). Although all extracts showed broad O–H stretching around 3417–3335 cm^−1^, reflecting phenolic and alcoholic groups, marked differences appeared in several key functional regions. Green, tubuna and plebeia propolis exhibited characteristic aliphatic C–H stretching at 2977–2934 cm^−1^, whereas tubuna presented an additional 2903 cm^−1^ band and a unique ketone signal at 1712 cm^−1^, distinguishing it from the carboxylic-acid bands (1695–1696 cm^−1^) found in green, plebeian and mandaçaia samples. Mandaçaia also displayed an intense 1634 cm^−1^ band linked to phenolic resins and antioxidant activity, together with a strong aromatic deformation at 1516 cm^−1^, which was absent in plebeia. Bands in the 1455–1442 cm^−1^ region (CH_2_/CH_3_ bending) and 1386–1380 cm^−1^ (methyl bending) contributed to differentiation, while the 1220–1200 cm^−1^ region, associated with phenolic resins, was particularly strong in mandaçaia. Distinctive C–O stretching bands also aided classification: 1168 cm^−1^ (tubuna, fatty acids), 1126–1124 cm^−1^ (green and tubuna), 1090–1091 cm^−1^ (mandaçaia) and 1048–1032 cm^−1^ (C–O of primary alcohols). The lower-frequency region revealed diagnostic markers: an α-glycosidic signal at 879 cm^−1^ in mandaçaia (glycosylated polyphenols), a diterpene marker at ~886 cm^−1^ in plebeia (labdane diterpenes) and a lipid-associated peak at 714 cm^−1^ in tubuna.

Quero et al. [15] applied ATR–FTIR to eight commercial liquid propolis extracts originating from Brazil, China, Colombia, New Zealand and United States, demonstrating that mid-IR functional-group patterns remain informative even in pre-processed products. All samples showed the characteristic broad O–H stretching band at 3350–3250 cm^−1^, reflecting phenolic and alcoholic groups, although its intensity varied according to extraction protocols (e.g., reduced in samples where alcohol solvent was removed). The 2980–2870 cm^−1^ region contained asymmetric and symmetric C–H stretching from aliphatic hydrocarbons, wax esters and flavonoids. Distinct C=O stretching vibrations at 1645–1635 cm^−1^ were associated with flavonoids, such as galangin and pinocembrin, as well as aliphatic aldehydes and ketones, and their presence or absence contributed to sample differentiation. A unique aromatic C=C band at 1511 cm^−1^ appeared only in one commercial extract (“1839”), indicating variations in aromatic flavonoid content. Additional discriminating signals included CH bending and aromatic deformation in the 1460–1450 cm^−1^ and 1370–1320 cm^−1^ regions, associated with methyl bending and O–H deformation in phenolic acids and flavonoids. The 1280–1249 cm^−1^ region reflected C–O–C stretching in esterified phenolics, while peaks between 1140 and 1100 cm^−1^ indicated C–N stretching, suggesting the presence of amino-acid derivatives or bee-secreted proteins. The strongest discriminating power, however, was observed in the 1090–1020 cm^−1^ region, corresponding to C–O stretching of primary alcohols, phenolic esters and glycosides; PCA identified 1017 cm^−1^ as the most influential wavenumber driving separation of samples. The 990–830 cm^−1^ aromatic C–H out-of-plane bending region further distinguished substitution patterns in flavonoids and phenolic compounds.

In the study by Nada et al. [30], UV spectra collected from 60 global propolis samples revealed clear type-dependent *λ*_max_ shifts that assisted in discriminating blue-, orange- and green-type samples. Blue-type propolis, which is enriched in flavonols such as quercetin, kaempferol and galangin, showed stronger absorptions around 350–365 nm, consistent with highly conjugated B-ring systems. Green-type propolis displayed characteristic bands at 296–300 nm, reflecting the presence of prenylated cinnamic acids, especially artepillin C and related derivatives known for strong absorbance near 300 nm. Orange-type propolis exhibited signals with *λ*_max_ between 325 and 345 nm, corresponding to elevated levels of caffeic acid, isoferulic acid, 3,4-dimethoxycinnamic acid, and other phenolic acids identified via HPTLC–MS. These compounds display π→π* transitions in this region and can be distinguished from the more flavonoid-rich blue type. Upon addition of AlCl_3_, Nada et al. observed significant bathochromic shifts (typically +20–40 nm), especially in samples rich in flavonols bearing ortho-dihydroxyl groups (quercetin, luteolin) and keto–hydroxyl systems capable of forming acid-stable complexes. AlCl_3_-induced shifts were strongest in blue-type samples, supporting their flavonoid content, while green-type samples showed minimal shifts due to the dominance of prenylated phenolic acids rather than flavonols.

NMR provides the most detailed structural insight among spectroscopic techniques and is capable of simultaneously detecting phenolics, terpenoids, lipids, sugars and aromatic resins in crude propolis extracts. Cuesta-Rubio et al. [48] demonstrated that ^1^H-NMR and ^13^C-NMR spectra of 65 Cuban samples formed three clear chemotypes: brown (BCP), red (RCP) and yellow (YCP), corresponding to distinct classes of secondary metabolites. Brown propolis exhibited intense aromatic AA′XX′Y spin systems at δ 7.26–7.65 and diagnostic isoprenyl methyls at δ 1.56–1.72, characteristic of polyisoprenylated benzophenones, such as nemorosone. Red propolis displayed strong methoxy signals at δ 3.75–3.82 and downfield aromatic protons at δ 6.3–8.1, associated with isoflavonoids (pterocarpans, isoflavans, isoflavones). Yellow propolis was dominated by aliphatic resonances at δ 0.7–2.1 with only weak downfield signals, consistent with aliphatic diterpenoids and long-chain lipophilic compounds. These patterns highlight the capacity of ^1^H-NMR to provide chemotype-level discrimination without prior chromatographic separation.

The study by Anđelković et al. [8] demonstrated that NMR signals can reliably encode ecological gradients when coupled with chemometrics. The authors combined NMR, IR, UV–Vis and O-PLS/O^2^PLS modeling to correlate Serbian poplar-type propolis with altitude. They found that phenolic glycerides, which show characteristic glycerol CH–O protons at δ 3.3–4.3 and ester carbonyl-adjacent methylenes at δ 2.2–2.5, were enriched in high-altitude samples, whereas flavonoids (pinocembrin, chrysin, galangin) dominated low-altitude extracts.

Pavlović et al. [11] illustrated that NMR and HPLC-Q-Orbitrap MS provide complementary structural information for European poplar-type propolis, especially for resolving isomeric flavonoids whose aromatic proton patterns differ subtly (e.g., H-6/H-8 in flavones at δ ~6.2–6.8 vs. H-2′/H-6′ in flavonols at δ 7.3–7.8). Papotti et al. [40] further used high-resolution ^1^H-NMR to classify Italian propolis obtained by three harvesting techniques, achieving 96.7% predictive accuracy using spectral regions from δ 4.5–13.0, with 2D HMBC providing additional structural confirmation.

Watson et al. [55] digitized ^1^H-NMR spectra of 43 samples from multiple continents and applied PCA, showing clear separation based on geographical origin. They demonstrated that aromatic regions (δ 5.5–8.5 ppm), especially phenolic and flavonoid signals, were the major drivers of clustering. Maraschin et al. [38] applied NMR-based metabolomics to 59 Brazilian samples and used PLS-DA and Random Forest to achieve >90% classification accuracy by season and agroecological zone. Their feature selection approach identified key discriminatory metabolites, including monosaccharides (δ 4.5–5.0), organic acids (δ 2.1–4.3), phenolics, and terpenoid resonances, showing that NMR captures climate-driven metabolic shifts.

Tiveron et al. [56] used ^1^H and ^2^D NMR to elucidate the structures of seven lignans and lignan precursors isolated from Brazilian certified organic propolis, providing the first report of several of these compounds in propolis. The most active subfractions, selected through bioassay-guided fractionation, yielded coniferyl alcohol, coniferyl aldehyde, lariciresinol, secoisolariciresinol, balajaponin D, pinoresinol and matairesinol, whose characteristic NMR features included benzylic methoxy and methylenic protons (δ 3.2–4.2), aromatic ABX patterns typical of guaiacyl phenylpropanoids (δ 6.6–7.2), and downfield aldehydic signals near δ 9.6 (coniferyl aldehyde). These lignans exhibited strong antioxidant activity and are proposed as new chemical markers for organic propolis sourced from southern Brazilian conservation areas.

### 4.3. Elemental Analysis

Studies show that multielement profiling can provide sensitive geographical markers in raw propolis, but extraction-related variables and soil heterogeneity must be considered when evaluating tinctures or processed propolis products.

Cantarelli et al. [10], as shown in Table S1, applied neutron activation analysis (NAA) to quantify 14 trace elements in 96 Argentinean raw propolis samples and assess their geographical origin. Significant regional differences were observed for Fe, Zn, Rb, Cr, Ce, La, Br, Sb, Sm, and Th, reflecting differences in local soils and vegetation. Chemometric analysis revealed that eight elements (Br, Co, Cr, Fe, Rb, Sb, Sm, Zn) were the strongest discriminators of provenance, as selected by stepwise LDA. LDA achieved ~98% correct classification, demonstrating that mineral fingerprints provide robust territorial markers for propolis authentication. These findings highlight that trace elements, even at μg·kg^−1^ levels, present important geographical signals and can complement organic–chemical markers in multi-parameter authentication models.

Soós et al. [41] examined the elemental composition of 27 Hungarian raw propolis samples and their corresponding 80% ethanol tinctures using ICP-OES/ICP-MS. The study characterized 36 elements and calculated transfer coefficients (TCs) to evaluate how efficiently elements migrate from raw propolis into tinctures. Essential minerals such as K, Na, P, Mg, B, Zn, Mn and Co showed the highest transfer rates (14–67%), while most potentially toxic elements (Al, Ba, Cd, Cs, Sr, V) transferred poorly (<10%). Nickel and chromium exhibited intermediate transfer efficiencies (Ni 7–63%, Cr 1–30%), identifying them as critical elements to monitor in raw propolis, especially when environmental contamination is possible. Strong correlations (r > 0.80) between raw propolis and tincture concentrations were observed for Na, Cr, S, Cu, P, Co, and K, indicating their potential use in predictive modeling. However, the authors demonstrated that element-based geographical authentication becomes unreliable after extraction, due to large sample-to-sample variation in transfer coefficients. This work reinforces that elemental profiles can discriminate raw propolis by origin, but extraction processes may weaken or distort these mineral signatures in consumer products.

### 4.4. Volatilomics

Volatile organic compounds (VOCs) represent a distinct feature for propolis characterization, while reflecting plant resin sources and environmental factors. Headspace-based GC–MS techniques, dynamic (DHS) and static (SHS), combined with olfactometry and electronic nose systems, have successfully differentiated propolis from diverse regions, as shown in Table S1.

Cheng et al. [57] combined HS–SPME–GC/MS and an electronic nose (E-nose) to discriminate Chinese propolis by geographical origin based on its volatile profile. A total of 57 volatiles were identified, dominated by monoterpenes (α-pinene, β-pinene, limonene), aromatic aldehydes, ketones and small esters, with substantial compositional differences among provinces. Propolis from northern China showed higher levels of α-pinene and camphene, whereas southern samples contained increased amounts of benzaldehyde, phenylacetaldehyde, and oxygenated terpenoids, reflecting differences in local flora and resinous plant species. PCA gave clear spatial clustering, while the E-nose classification closely matched the GC/MS results, confirming that volatile terpenes and aromatic compounds encode strong regional signals.

de Oliveira Sartori et al. [28] investigated the volatile fraction of organic and non-organic brown propolis from southern Brazil using SHS–GC/MS, identifying 99 volatiles dominated by monoterpenes and sesquiterpenes. α-Pinene was the most abundant compound in nearly all samples (up to 68–90%), followed by β-pinene, β-eudesmol, γ-eudesmol, trans-verbenol, α-campholenal and other resin-derived terpenoids. Volatile profiles of propolis overlapped strongly with those of *Araucaria angustifolia* and *Pinus* spp. resins, and PCA showed that propolis from the General Carneiro region clustered closely with *Araucaria resin*, providing compelling evidence for its botanical origin. Heatmap analysis further revealed sample-specific signatures, including furfural enrichment in non-organic propolis and α-bisabolol enrichment in organic samples.

Wang et al. [44] used nanoESI-MS to obtain rapid volatilome-adjacent molecular fingerprints (<30 s analysis time) of 37 Chinese propolis samples, identifying 66 small-molecule compounds, including phenolic acids (caffeic, p-coumaric, cinnamic acids), caffeic-acid esters (CAPE, drupanin), flavonoids (pinobanksin derivatives) and small aromatic volatiles detectable in negative ion mode. Distinct *m*/*z* patterns allowed discrimination between ethanol extracts and water extracts, with ethanol showing higher intensities of flavonoids and esters (*m*/*z* 247, 283, 313), while water contained more free phenolic acids (*m*/*z* 179). Multivariate modeling (PLS-DA) clearly separated propolis from different climate zones (temperate, subtropical) and colors (yellow, brown, black, red). Differential metabolites included benzoic acid, 2-methoxyphenol, sinapinic acid, 3,4-dimethoxyhydrocinnamic acid, desmosflavone, and pinobanksin-3-O-acetate, which acted as chemotaxonomic and color-specific markers. Machine-learning models (SVM, RF, LR, GB) achieved classification accuracies of 85–96%, demonstrating that rapid direct-MS fingerprints offer a promising approach for propolis origin and color authentication when volatile-rich GC/MS profiling is not feasible.

### 4.5. Proteomics

Shahali et al. [58] introduced proteomics as a novel analytical dimension for propolis characterization, applying SDS-PAGE, MALDI-TOF-MS and LC-ESI-MS to identify 74 non-redundant proteins in Belgian and Iranian poplar-type propolis. The majority of detected proteins originated from *Populus* spp., including abundant pathogenesis-related (PR) proteins such as β-1,3-glucanases (PR-2), endochitinases (PR-8), PR-4 chitin-binding proteins, thaumatin-like proteins (PR-5) and non-specific lipid-transfer proteins (PR-14). These plant defense proteins reflect the protective biological role of resin exudates and may contribute to the antimicrobial activity typically associated with poplar-type propolis. In addition, bee-derived proteins—including major royal jelly proteins (MRJP 1-3, 5) and the venom allergen Api m 1—were detected, providing insight into allergenicity and the proteinaceous contributions of *Apis mellifera*. Total ion chromatograms revealed shared proteomic fingerprints between Belgian and Iranian propolis samples, supporting a common *Populus* origin. The study demonstrates that proteomic markers can complement chemical profiling and may offer new opportunities for botanical origin verification and allergen identification in propolis.

### 4.6. Electroanalytical Techniques

Ristivojević et al. [59] demonstrated the utility of cyclic voltammetry (CV) as a rapid, green, and cost-effective electroanalytical tool for propolis authentication. Using a glassy carbon electrode and phosphate buffer (pH 7), they recorded anodic and cathodic voltammograms of 46 Serbian poplar-type propolis samples and extracted key redox parameters, including oxidation peak potentials (Epa), reduction peak potentials (Epc), peak currents (Ipa/Ipc), and charge passed (Q600). These voltammetric fingerprints revealed characteristic multi-step oxidation patterns associated with phenolic constituents, enabling clear discrimination between orange-type (*Populus nigra*) and blue-type (*Populus alba*) propolis. Orange samples exhibited higher anodic peak currents and larger Q600 values, consistent with their higher total phenolic and flavonoid contents, whereas blue samples showed additional oxidation peaks at more positive potentials. As a non-destructive, low-solvent and high-throughput technique, electroanalytical profiling provides a valuable complementary approach for propolis quality control and authenticity assessment.

### 4.7. Palynology

Although pollen analysis alone cannot unambiguously determine the botanical origin of propolis, it provides important contextual information on the vegetation surrounding the apiary and often supports chromatographic and spectroscopic classification. It is often limited by the fact that bees collect resin from leaf and bud exudates and not necessarily from pollinating flowers, but when interpreted together with chemical fingerprints, palynology can help trace dominant plant sources and detect regional floral signatures that influence propolis composition.

Güzelmeriç et al. [27] reported that Turkish propolis contained abundant pollen grains from *Fabaceae*, *Lamiaceae*, *Rosaceae*, *Castanea sativa* and *Salix* spp., with several families exceeding the dominant pollen threshold (>45%). These taxa are typical components of the Anatolian flora and were consistent with the phenolic fingerprints indicating poplar-type propolis. Notably, *Castanea sativa* pollen was particularly abundant in some O-type samples and correlated with their strong phenolic and antioxidant profiles, reflecting the co-occurrence of chestnut forests and *Populus nigra* vegetation in several Turkish regions.

Kolaylı et al. [4] also characterized Anatolian propolis and documented a much broader pollen spectrum, including *Pinaceae*, *Quercus* spp. and *Helianthus annuus*, confirming its multifloral nature. Their findings emphasize that even when propolis chemistry is dominated by *Populus* resins, bees forage from a wide range of nectar and non-nectar plants, producing diverse pollen assemblages that reflect both local flora and seasonal variability.

Nada et al. [30] identified 28 pollen types from 13 botanical families in Egyptian propolis, with *Asteraceae* being the most abundant. The absence of dominant pollen types in all samples indicated a strictly multifloral origin. Interestingly, *Eucalyptus* pollen appeared frequently, consistent with the prevalence of *Eucalyptus camaldulensis* in Egyptian landscapes and with the unique flavonoid/phenolic patterns observed by HPTLC and UV–Vis chemometrics in the same study.

## 5. Chemometrics and Machine Learning for Data Treatment

Chemometrics and machine learning have become essential tools in authentication strategies, enabling objective data interpretation and classification based on large, multidimensional analytical datasets. By integrating chemical fingerprints from chromatographic, spectroscopic and other platforms, these computational methods allow discrimination of propolis types by botanical source, geography, and even climate or bee species. Unsupervised methods are primarily applied for exploratory data analysis, revealing natural sample groupings and chemical relationships without predefined class labels, while supervised models are used for predictive classification once categories are known. While early studies relied primarily on unsupervised exploratory multivariate statistics, modern approaches incorporate supervised models and machine learning algorithms for predictive authentication and pattern recognition. Beyond qualitative discrimination, chemometric regression models enable quantitative prediction of marker compounds and functional properties from spectral or chromatographic fingerprints.

**Figure 4:**
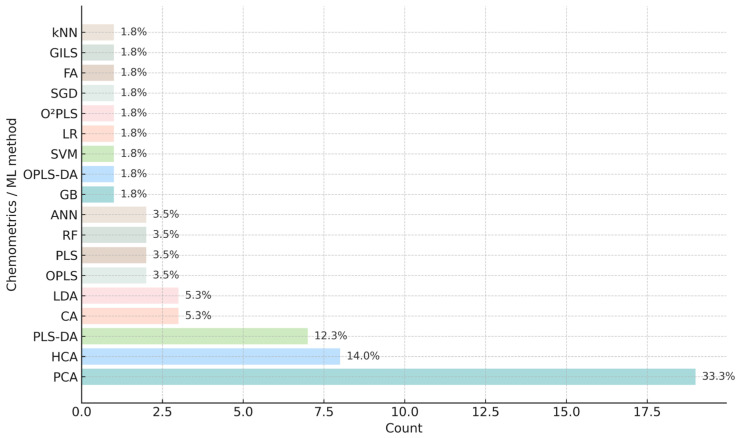
Distribution of chemometric and machine-learning techniques applied in 43 propolis authentication studies (2005–2025) from Table S1.

To provide a quantitative overview of chemometric and machine-learning (ML) tools used across global propolis authentication studies, we analysed the entries reported in Table S1 (Figure 4). Unsupervised approaches clearly dominate current practice: PCA and FA together represent 35.1% of all applications, while CA and HCA account for an additional 19.3%. These methods remain foundational for exploratory pattern recognition, outlier detection and preliminary sample grouping. Among supervised techniques, PLS-DA (12.3%) is the most frequently applied, followed by LDA (5.3%), PLS and OPLS (3.5% each). ML-based classifiers, including ANN, RF, SVM, kNN and others, collectively represent 20.3% of all applications, demonstrating a growing interest in predictive modelling and non-linear pattern recognition.

### 5.1. Unsupervised, Supervised and Regression Chemometric Tools

Chemometric analyses bridge analytical chemistry and data science, transforming large sets of spectral or chromatographic signals into interpretable chemical information. PCA and HCA remain the most widely employed exploratory tools. Early ^1^H-NMR-based work by Watson et al. [55] demonstrated that PCA of spectral data effectively clustered 43 propolis samples by geographical origin. PCA has since been applied across multiple analytical platforms: FTIR spectral datasets successfully distinguished Malaysian stingless bee propolis by locality [53] and discriminated commercial extracts by origin [14], while phenolic-composition matrices separated European poplar-type propolis from Turkish and Uruguayan variants [60]. In addition, PCA of LC–MS and UV–Vis data revealed seasonal clustering and bioactivity-related shifts in red propolis [43]. Unsupervised analyses have also been used to explore volatile and elemental patterns: dynamic headspace–GC/MS coupled with PCA grouped Chinese propolis into four regional chemotypes [57], and PCA of ICP-derived trace-element data identified distinct clusters of Hungarian and Argentine samples [11,45]. Beyond PCA and HCA, Sârbu and Moţ [42] introduced fuzzy divisive hierarchical clustering of TLC fingerprints, which managed to capture subtle differences between Romanian meadow- and forest-type propolis. Together, these unsupervised models facilitate visualization of compositional variability and preliminary identification of discriminating chemical factors.

Once class membership is defined, discriminant algorithms are applied to construct predictive authentication models. LDA, partial least squares discriminant analysis (PLS-DA), orthogonal PLS (O-PLS and O^2^PLS), stepwise LDA and kNN are the most frequently reported tools. Cantarelli et al. [10] achieved 98% correct regional classification of 96 Argentinian propolis samples using neutron-activation elemental data and stepwise LDA combined with kNN. Anđelković et al. [8] integrated NMR, IR and UV spectral data with O-PLS/O^2^PLS modeling to correlate poplar-type propolis composition with altitude, identifying phenolic glycerides and flavonoids as altitude-specific markers. In Brazil, do Nascimento et al. [43] used PLS-DA and O-PLS-DA to relate seasonal variations in flavonoids and guttiferones to antioxidant and antibacterial activities in red propolis, demonstrating the capacity of chemometric models to link composition with function. Similar supervised strategies have been applied to FTIR [54], HPTLC [27], and LC–MS datasets [36], enabling reliable classification by botanical or geographical origin.

Partial least squares (PLS) regression and genetic inverse least squares (GILS) have proven particularly effective for modeling linear relationships between spectral intensity and analyte concentration. Güzelmeriç et al. [45] applied both methods to digitized HPTLC images, achieving accurate quantification of phenolic markers, such as caffeic acid and pinocembrin directly from chromatographic fingerprints, demonstrating the feasibility of reagent-free quantitation in propolis quality control. OPLS has extended multivariate calibration beyond composition toward bioactivity prediction. Nada et al. [30] combined digitally enhanced HPTLC and UV–Vis spectroscopic data with OPLS modeling to link chemical fingerprints to α-glucosidase and α-amylase inhibition activities. The model identified the key efficacy markers, highlighting how regression-based chemometrics can connect composition with biological functionality. Correlative multivariate approaches have also been employed to explore climate–metabolite–activity relationships. Do Nascimento et al. [43] integrated PCA-X, PLS-DA, and OPLS-DA with VIP scoring to reveal that seasonal variations in red Brazilian propolis were driven by fluctuations in guttiferones and flavonoids, which correlated strongly with antioxidant and antibacterial activities. Similarly, Pearson correlation and VIP analysis have been used to identify compositional drivers of bioactivity in both tropical and temperate propolis types [43,54].

### 5.2. Machine Learning Algorithms

Recent studies have expanded beyond traditional chemometrics toward modern ML and artificial intelligence (AI) approaches, which enhance classification power and predictive accuracy of large datasets. Supervised ML algorithms, such as SVM, RF, ANN, and decision trees are increasingly used for propolis classification.

Maraschin et al. [38] combined NMR-based metabolomics with PLS-DA and RF models to classify Brazilian propolis by season and agroecological zone, achieving over 90% accuracy and identifying climate-dependent metabolites that influence bioactivity. Pant et al. [51] trained an ANN on LC-ESI-QTOF-MS datasets to predict antioxidant capacity from total phenolic and flavonoid contents, identifying CAPE, galangin and β-carotene as the most influential predictors and outperforming conventional regression models. Wang et al. [44] employed nanoESI–MS combined with PLS-DA and ANN classifiers to discriminate 37 Chinese propolis samples by climate zone and color, achieving 95.6% and 85.3% predictive accuracy, respectively, and demonstrating the potential for real-time classification. Feature-selection procedures within RF and SVM models have helped identify discriminating compounds, such as artepillin C, guttiferones, liquiritigenin and other flavonoids that drive sample differentiation [44].

As open-access metabolomics repositories, such as MetaboLights [61], and large community-curated MS platforms, such as GNPS [62], continue to expand, ML models trained on harmonized, well-annotated datasets will increasingly support cross-study comparability and global standardization of propolis chemotypes. The availability of standardized metadata, controlled vocabularies and shared libraries will enable the development of more robust, transferable predictive models that are not limited to a single laboratory or analytical platform. At the same time, the adoption of explainable AI approaches (e.g., SHAP values, permutation importance) will clarify the specific contribution of individual metabolites and spectral regions to model decisions, improving transparency, reproducibility and regulatory acceptance. Thus, the integration of data-driven frameworks will provide a pathway toward consensus marker panels, predictive origin-authentication algorithms and internationally harmonized propolis quality-assurance standards.

## 6. Geographical Case Studies

The chemical diversity of propolis is closely linked to regional flora, climatic conditions and bee species, giving rise to distinct chemotypes across the world. Comparative authentication studies have revealed that specific marker compounds, spectral signatures or volatile profiles can reliably indicate both the botanical and geographical origin of propolis. The following case studies, shown in Table 2, summarize representative findings across major producing regions.

**Figure 5:**
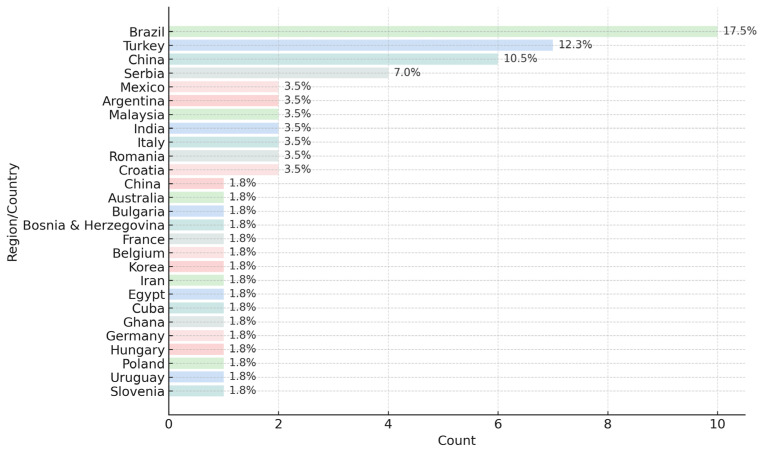
Distribution of propolis authentication studies by geographical origin in 43 propolis authentication studies (2005–2025) from Table S1.

The geographical distribution of published propolis authentication studies shows clear regional clustering, reflecting both the research activity of individual countries and the global diversity of propolis chemotypes—Table S1. As illustrated in Figure 5, Brazil, Turkey and China dominate the literature, together accounting for more than 40% of all collected studies. This is consistent with their long-standing involvement in propolis research, particularly Brazil, where diverse plant sources give rise to multiple chemotypes (green, red, brown). Countries such as Serbia, Mexico, Argentina, Malaysia, India, Italy, Romania and Croatia form a second level of research activity, each contributing between 3 and 4% of the total dataset. A wide range of countries, including Australia, Bulgaria, Poland, Slovenia, France, Korea, Iran, Egypt, Uruguay, Cuba, Ghana, Germany and others, appear with lower frequency, usually representing isolated case studies or emerging research groups. The dataset demonstrates extensive global interest, but with strong concentration in a few highly active regions. This uneven distribution highlights the need for broader geographical representation, particularly in regions where propolis types remain chemically undercharacterized or lacking standardised authentication workflows.

### 6.1. Europe

European propolis is dominated by the poplar type, characterized by flavonoids and phenolic esters derived from *Populus nigra* and *P. tremula* resins. Studies across Romania, Croatia, Hungary, Turkey and Serbia consistently show the prevalence of pinocembrin, chrysin, galangin and CAPE as the defining markers [4,8,27,36,59,63].

In Romania, the authors successfully differentiated meadow- and forest-origin propolis, with marker zones corresponding to caffeic acid derivatives and flavones (Table 2) [52]. Croatian propolis tinctures, revealed that polyphenolic profiles can serve for regional traceability and routine quality control, as shown in Table 2 [64].

Turkish propolis exhibited three subtypes: orange (O, *P. nigra*), blue (B, *P. tremula*) and a non-phenolic type [36]. The O-type displayed the richest phenolic and flavonoid content and highest antioxidant activity, with quercetin, pinobanksin, CAPE and galangin as discriminating markers. In Serbian and Hungarian samples, studies confirmed poplar-type phenolics as major constituents, with chrysin, pinocembrin and caffeic acid as quality markers. European propolis is thus generally chemically homogeneous, reflecting its shared poplar origin, yet subtle geographic differences can be resolved through multivariate models.

### 6.2. Latin America

Latin American propolis exhibits remarkable chemical diversity, spanning the green, red and brown Brazilian types and distinct Cuban varieties. Brazilian green propolis, sourced from *Baccharis dracunculifolia*, is rich in prenylated cinnamic acids, such as artepillin C and baccharin signature compounds responsible for its antioxidant and antimicrobial activities [37]. Red propolis, derived from *Dalbergia ecastophyllum*, contains abundant isoflavonoids (formononetin, liquiritigenin, naringenin) and guttiferones that fluctuate seasonally with climate [43]. Brown propolis from *Araucaria angustifolia* regions contains sesquiterpenes and monoterpenes, traceable through volatile fingerprinting, as shown in Table 2 [28].

Recent work on stingless bee propolis from southeastern Mexico demonstrated that phenolic composition and bioactivity can serve as reliable geographical classifiers [31]. Samples of *Melipona beecheii* collected from six Mexican states showed strong regional differences in total phenolic and flavonoid contents, confirming that phenolic fingerprints coupled with bioactivity assays can discriminate propolis from distinct vegetation zones. Recent findings from Brazil further highlight the diversity of both stingless bee and honeybee propolis [54,65]. Surek et al. [54] evaluated green *Apis mellifera* propolis alongside tubuna, mandaçaia, and plebeia propolis from *Meliponini* species, revealing clear differences among propolis types, and could reliably discriminate Brazilian stingless bee propolis. The authentication was performed with MIR spectral profiles coupled to chemometrics, and driven by variations in phenolic acids, flavonoids, prenylated cinnamic acids, diterpenes and fatty acids. The 39 brown propolis samples collected by honeybees from three regions of Paraná (South, East, West) were investigated using GC–MS to characterize their chemical profiles [65]. Resinic diterpenes, aromatic acids and various volatile components were identified as major constituents, with PLS-DA analysis showing clear regional clustering and strong chemical differentiation among propolis from different areas of the state.

Cuban propolis is classified into three types: brown, red, and yellow based on metabolite composition, as shown in Table 2 [48]. Brown Cuban propolis contains polyisoprenylated benzophenones; red Cuban propolis is dominated by isoflavonoids; and yellow Cuban propolis shows aliphatic compounds. Latin American propolis types are chemically distinct and serve as benchmarks for non-poplar chemotypes worldwide.

### 6.3. Asia

Asian propolis is highly heterogeneous, reflecting diverse vegetation and climatic gradients. In China, 12 regional samples were discriminated based on acids, esters, terpenes and aromatics, identifying benzyl p-coumarate and p-coumaric acid as diagnostic for Changbai Mountains propolis, as shown in Table 2 [49,57]. Organic-specific marker sets of flavonoids and phenolic acids further differentiated Chinese, Brazilian and Korean propolis [29,66].

In Indian propolis, region-specific biomarkers, proanthocyanidins, quinate derivatives, carotenoids and phytosterols, were identified as discriminating features [51]. Malaysian stingless bee propolis from *Geniotrigona thoracica* exhibited unique terpenoid-rich profiles, as shown in Table 2 [53].

### 6.4. Africa

African propolis remains less studied, but recent research from Ghana demonstrated that solvent type and source significantly influence its composition and biological activity. Ghanaian propolis profiling revealed caffeic acid, flavonoid derivatives and silacyclononanone as the main discriminating compounds, as shown in Table 2 [46]. PCA and HCA showed that ethanolic–water extracts exhibited the highest antioxidant and α-amylase inhibitory activity, correlating with phenolic abundance.

### 6.5. Australia

The emerging Australian propolis industry has revealed distinctive volatile and phenolic profiles influenced by unique native flora [67]. Analysis of 158 samples identified up to 16 chemotypes, with high total phenolic content and potent antioxidant activity, as shown in Table 2. The study effectively discriminated between eastern and western coastal propolis, indicating strong geographical influence. Volatile composition studies revealed terpenoid-dominated profiles distinct from both European and Brazilian types, underscoring Australia’s unique propolis chemistry and commercial potential.

**Table 2:** Some examples of case studies on authentication of geographical origin across continents (Europe, Asia, Africa and Australia).

Propolis Type	Region/Country	Bee Type	N Samples	Analytical Methods	Target Markers	Chemometrics/ML	Authentication Target	Key Outcomes	Ref
Poplar-type	Turkey	*A.m. caucasica; A.m. anatolica; A.m. carnica*	3	GC–MS	48 compounds; *Populus* and *Salix* markers	-	Bee species origin	Bee race behavior differences influence propolis composition	[52]
Poplar-type	Croatia	*Apis mellifera*	6	HPLC; HPTLC; GC-MS; UV-Vis	Flavonoids; Phenolic acids	-	Geographical origin; QC	HPTLC, HPLC and GC characterization and QC	[64]
Chinese poplar-type	China	*Apis mellifera*	12	DHS-GC/MS; E-nose; GC-O	99 volatiles; odor-active compounds	PCA	Geographical origin	Geographical regions classified; key odorants identified	[57]
Mixed-type, Multifloral	India	*Apis mellifera*	30 (Himachal Pradesh, Punjab, Haryana, Rajasthan)	LC-ESI-QTOF-MS; RP-HPLC; TPC; TFC; DPPH/FRAP	beta-Carotene; Galangin; CAPE	PCA; ANN	Geographical origin	Regional characterization of northern Indian propolis	[51]
Stingless bee propolis (*Geniotrigona thoracica*)	Malaysia	Stingless bee	5	HPTLC; FTIR	Flavonoids; Phenolics; Terpenoids	PCA; HCA	Geographical origin	Three clusters by location; FTIR + chemometrics classify effectively	[53]
Mixed-type	Ghana	*Apis mellifera*	3	TPC; TFC; DPPH; TLC	Caffeic/quinic derivatives; Quercetin; Naringenin; Hesperidin; Rosmarinic acid; Methyl cinnamate; Steroids; Triterpenoids	PCA; HCA; ANOVA	Source/solvent differentiation	Regional and solvent authentication	[46]
Brazilian brown	Brazil	*Apis mellifera*	7	SHS-GC-MS	Monoterpenes; Sesquiterpenes	PCA; HCA; heatmap	Botanical origin (resin source)	Volatile profile matched Araucaria angustifolia resins; strong clustering	[28]
Brown; Red; Yellow	Cuba	*Apis mellifera*	65	HPLC-PDA; LC-MS; ^1^H NMR; ^13^C NMR	Polyisoprenylated benzophenones; Isoflavonoids; Pterocarpans	-	Type classification (color-based)	Three chemical types: brown = benzophenones; red = isoflavonoids; yellow = aliphatic	[48]
Stingless bee (Melipona beecheii)	Mexico	*Melipona beecheii*	35	UV-Vis (TPC; TFC)	Total phenolics; Total flavonoids	PCA; HCA	Geographical origin	Clustered samples by region and bioactivity	[31]
16 high-grade types	Australia (different regions)	*Apis mellifera*	158	HPLC-UV; ^1^H NMR; DPPH assay	Phenolics; Flavonoids (chrysin; pinocembrin; galangin; prenylated stilbenes; artepillin C)	PCA; PLS-DA	Geographical origin; QC	Identified 16 high-grade types; several exceeded Brazilian green/red propolis in antioxidant capacity	[67]

Caffeic acid phenethyl ester (CAPE); quality control (QC); thin-layer chromatography (TLC); high-performance thin-layer chromatography (HP-TLC); hierarchical cluster analysis (HCA); high-performance liquid chromatography with ultraviolet detection (HPLC-UV); liquid chromatography–mass spectrometry (LC-MS); gas chromatography-mass spectrometry (GC-MS); dynamic headspace (DH); static headspace (SH); gas chromatography-olfactometry (GC-O); ultraviolet–visible spectroscopy (UV/Vis); fourier transform infrared spectroscopy (FTIR); 2,2-diphenyl-1-picrylhydrazyl (DPPH); principal component analysis (PCA); partial least squares discriminant analysis (PLS-DA); nanospray electrospray ionization mass spectrometry (nanoESI-MS); liquid chromatography–electrospray ionization quadrupole time-of-flight mass spectrometry (LC-ESI-QTOF-MS); reversed-phase high-performance liquid chromatography (RP-HPLC); total phenolic content (TPC); total flavonoid content (TFC); ferric reducing antioxidant power assay (FRAP); artificial neural network (ANN); proton nuclear magnetic resonance spectroscopy (^1^H NMR); carbon 13 nuclear magnetic resonance spectroscopy (^13^C NMR); high-performance liquid chromatography coupled with a photodiode array detector (HPLC-PDA).

## 7. Regulatory and Standardization Perspectives

Despite the expanding global market for propolis-based products, the absence of unified quality and authenticity standards remains a major challenge. Unlike honey, whose composition, labeling, and purity are regulated under the Codex Alimentarius [68] and the EU Honey Directive [69], propolis lacks harmonized international criteria defining its botanical origin, chemical markers or acceptable compositional ranges. The result is significant variability in propolis supplements and extracts sold globally, often leading to inconsistent bioactivity, adulteration, and difficulties in cross-border trade. Establishing internationally accepted standards analogous to those for honey would provide a framework for quality assurance and consumer protection.

In Europe, no continent-wide propolis standard currently exists. Most EU countries rely on national pharmacopoeial or food-supplement guidelines that vary in defining minimum total phenolic content or solvent purity. Croatia represents one of the earliest examples of a structured approach: Medić-Šarić et al. [64] proposed a quality specification for propolis tincture, encompassing parameters such as polyphenolic content (via HPLC/HPTLC), solvent composition and microbiological purity. This framework, though not yet institutionalized by the EU, provides a model for national or regional adaptation. Similarly, studies from Romania, Turkey and Serbia highlight the need for reproducible analytical benchmarks to control the growing diversity of local propolis types [4,27].

In Latin America, regulatory progress is more advanced, particularly in Brazil, where propolis has achieved semi-formal recognition as an “apiceutical product.” The Brazilian Ministry of Agriculture (MAPA) and National Health Surveillance Agency (ANVISA) have introduced national guidelines defining categories, such as green, red and brown propolis, linking them to their botanical sources (*Baccharis dracunculifolia*, *Dalbergia ecastophyllum*, *Araucaria angustifolia*). Certified organic Brazilian propolis is subject to compositional verification and residue control, with lignans such as lariciresinol and matairesinol recognized as new authentication markers [56].

Other countries have also initiated regional traceability protocols. In Cuba, chemical classification of red, brown and yellow propolis based on NMR and LC–MS profiles laid the groundwork for national quality control [48]. Similarly, in Argentina, Cantarelli et al. [10] used multielement fingerprinting and discriminant analysis to propose a traceability scheme.

At the global level, the development of propolis standards will likely follow a quality-control paradigm similar to honey and other apicultural products: (i) rapid screening by spectroscopy or HPTLC coupled with chemometrics for preliminary classification; (ii) confirmatory analysis using LC–MS, NMR or GC–MS for quantification and marker verification; (iii) periodic cross-validation using proteomic, elemental, or volatile profiles to ensure long-term consistency.

Adopting this harmonized, data-driven approach could facilitate the creation of Codex-like propolis guidelines that define accepted chemical ranges, marker compounds and testing methods. Such standards would enhance traceability, reduce fraud, and support the establishment of Protected Designation of Origin (PDO) and Protected Geographical Indication (PGI) labels for high-value regional propolis, similar to those used for honey and olive oil. International collaboration between research networks, regulatory bodies and apicultural industries will be critical to achieve these goals, ensuring that propolis authentication evolves from laboratory practice to globally recognized policy.

## 8. Conclusions and Future Directions

Propolis authentication is of essential importance for ensuring product quality, consumer safety and trust. However, its chemical variability, caused by the bee species, regional flora, climate and extraction procedures, continues to challenge standardization. Reliable authentication therefore depends on analytical methods that are both validated and rapid, allowing fast and efficient screening and reproducible classification between different laboratories.

The most promising strategy combines multiple markers, different analytical techniques and chemometric data processing tools. For example, integrating chromatographic and spectroscopic data with interpretable machine-learning models can provide transparent and reproducible authentication. Such multi-omics and data-driven frameworks have the potential to identify marker sets for each major chemotype and to support the creation of shared spectral libraries for FTIR, NMR, HPTLC, HPLC and GC fingerprints. Furthermore, portable spectroscopic instruments, such as handheld FTIR, NIR or Raman devices equipped with embedded PCA or LDA models, offer promising opportunities for rapid in-field screening.

Future work should focus on establishing open and interoperable online databases that contain chemical profiles, biological activity and geographical information, and that can support the establishment of international guidelines and facilitate the recognition of regional propolis through PDO or PGI certification.

## Figures and Tables

**Table 1:** Summary of some representative studies on propolis (*Apis mellifera* bee) authentication (2005–2025), using chromatographic (HPLC, TLC), spectroscopic (NMR) and other approaches (palynology).

Propolis Type	Region/Country	N Samples	Analytical Methods	Target Markers	Chemometrics/ML	Authentication Target	Key Outcomes	Ref
Blue; Orange	Germany	64	HPTLC; DART-MS	Caffeic acid; Naringenin; Apigenin; Quercetin; Kaempferol; Galangin; Chrysin; Ellagic acid	PCA; HCA; LDA	Subtype discrimination	Blue type vs. Orange type discrimination	[3]
Poplar-type	Italy	60	HR-NMR	Flavonoids; Phenolic acids	Factor Analysis; General Discriminant Analysis	Harvesting method	^1^H NMR (4.5–13 ppm) classified by harvesting method with 96.7% predictive capacity	[40]
Orange; Blue; Non-phenolic	Turkey; Serbia	60	HPTLC; Palynology; UV-Vis; Antioxidant assays	Quercetin; Caffeic acid; CAPE; Pinobanksin; Galangin	PCA	Subtype discrimination; Geographical origin	Orange subtype richest; Turkish vs. European	[27]
Orange; Blue	Turkey	48	UHPLC-Orbitrap-MS/MS	Chrysin; Galangin; Pinocembrin; CAPE	ANOVA	Subtype classification	Orange subtype highest phenolics	[36]
Poplar-type tinctures	Hungary	252	ICP-OES/ICP-MS	Essential/Toxic elements	Correlation analysis	Elemental QC; Geographical origin	Geographical authentication impracticable	[41]
Romanian poplar-type	Romania	39	TLC; Image Analysis	Phenolic band patterns	Fuzzy clustering; PCA	Geographical origin; Botanical origin	Meadow area vs. Forest area	[42]
Red propolis	Brazil	39	UV-Vis; HPLC-DAD; LC-MS; DPPH	Flavonoids; Isoflavonoids; Polyprenylated Benzophenones	Correlation analysis; PCA; PLS-DA; OPLS-DA	Climate effect	Climate–metabolite relationship	[43]
Poplar-type	Argentina	96	NAA	Trace minerals	PCA; LDA; kNN	Geographical origin	Elemental fingerprints for specific provenance	[10]
Poplar-type	Mexico	12	^1^H-NMR; HPLC-UV-DAD	Pinocembrin; Pinobanksin; Chrysin; Galangin; Kaempferol; Quercetin; p-Coumarc acid; Naringenin	PCA; HCA	Botanical origin	Botanical sources: *Populus fremontii*; *Ambrosia ambrosioides*; *Bursera laxiflora*	[1]
Chinese poplar-type	China	37	nanoESI-MS; UPLC-MS/MS	Caffeic acid; p-Cinnamic acid; CAPE; Pinocembrin; Genistein; Ctric acid; Arctopicrn; Sinapinic acid; Benzoic acid; Gluconic acid; Quinic acid	PLS-DA; ANOVA; VIP Analysis; ML (RF, SVM, NN, LR, GB, SGD, Tree)	Climate-zone origin	Climate-zone and propolis-color authentication	[44]
Blue; Orange; Green	Egypt	60	HPTLC-ESI-MS; UV; Palynology	3,4-Dimethoxycinnamic acid; Caffeic acid; Isoferulic acid; Rosmarinic acid; Quercetin	OPLS; PLS	Type discrimination; bioefficacy markers	HPTLC fingerprinting of 3 global propolis types; Palynological identification of 13 plant families	[30]

Caffeic acid phenethyl ester (CAPE); ultra-high-performance liquid chromatography coupled to Orbitrap tandem mass spectrometry (UHPLC/Orbitrap/MS/MS); inductively coupled plasma optical emission spectrometry (ICP-OES); inductively coupled plasma mass spectrometry (ICP-MS); quality control (QC); thin-layer chromatography (TLC); direct analysis in real time mass spectrometry (DART-MS); hierarchical cluster analysis (HCA); linear discriminant analysis (LDA); high-performance liquid chromatography with diode-array detection (HPLC-DAD); liquid chromatography–mass spectrometry (LC-MS); 2,2-Diphenyl-1-picrylhydrazyl (DPPH); partial least squares discriminant analysis (PLS-DA); orthogonal projections to latent structures discriminant analysis (OPLS-DA); neutron activation analysis (NAA); k-nearest neighbor (kNN); proton nuclear magnetic resonance spectroscopy (^1^H-NMR); nanospray electrospray ionization mass spectrometry (nanoESI-MS); ultra-performance liquid chromatography tandem mass spectrometry (UPLC-MS/MS); variable importance in projection analysis (VIP Analysis); machine learning (ML); random forest (RF); support vector machine (SVM); neural network (NN); logistic regression (LR); gradient boosting (GB); stochastic gradient descent (SGD); decision tree algorithm (Tree).

## Data Availability

The raw data supporting the conclusions of this article will be made available by the authors on request.

## Supplementary Materials

The following supporting information can be downloaded at https://www.mdpi.com/article/10.3390/foods14244259/s1, Table S1: Summary of studies on propolis authentication (2005–2025).
