# Supplementary material for: Authentication of Propolis: Integrating Chemical Profiling, Data Analysis and International Standardization—A Review
Source: Foods. 2025 Dec 10;14(24):4259. doi: 10.3390/foods14244259 (PMC12732287; doi:10.3390/foods14244259)
Supplement: Supplementary file 1 [file foods-14-04259-s001.zip › foods-3999452-supplementary.pdf]

Supplementary material

Table S1. Summary of representative studies on propolis authentication (2005–2025).

Propolis type	Region/ Country	Bee type	N samples	Extraction solvent/ pretreatment	Analytical methods	Target markers	Chemometrics /ML	Authentication target	Bioactivity link	Key outcomes	Year*	Ref
Orange; Blue; Non-phenolic	Turkey; Serbia	*Apis mellifera*	60 (48 from Turkey; 12 from Serbia)	Chloroform; ultrasonication	HPTLC; Palynology; UV-Vis; Antioxidant assays	Quercetin; Caffeic acid; CAPE; Pinobanksin; Galangin	PCA	Subtype discrimination; Geographical origin	Antioxidant	Orange subtype richest; Turkish vs European	2018	[26]
Orange; Blue	Turkey	*Apis mellifera*	48 (27 orange; 17 blue; 4 unknown)	Ethanol 96%	UHPLC-Orbitrap-MS/MS; TPC; TFC; DPPH/ABTS; Antimicrobial	Chrysin; Galangin; Pinocembrin; CAPE	ANOVA	Subtype classification	Antioxidant; Antibacterial	Orange subtype highest phenolics	2018	[36]
Mixed-type	Turkey	*Apis mellifera*	4 (north, south, east and west of Turkey)	Water (deionized); Ethanol (ultra-pure)	HPLC-DAD; GC-MS	Caffeic acid; CAPE; Chrysin; Pinocembrin; Galangin	-	Chemical profiling	Antioxidant	Ethanolic extract richer in flavonoids vs water extract	2021	[19]
Mixed-type	Turkey (7 regions)	*Apis mellifera*	40	Ethanol 70%	RP-HPLC-PAD; Palynology	CAPE; Caffeic acid; Chrysin; Pinocembrin; Cinnamic acid	-	Botanical & geographical origin	Antioxidant	Major pollen sources Pinaceae, Castanea, Salix, Fabaceae; rich phenolic/flavonoid content	2023	[4]
Black Sea	Turkey	*Apis*	47	Ethanol 80%	HPTLC;	Caffeic acid;	PLS;	Quantitative	-	HPTLC	2023	[40]

poplar-type	(Black Sea region)	*mellifera*	(western, central, eastern Black Sea)		HPLC	Chrysin; CAPE; Galangin; Quercetin	GILS	QC / chemical type		images + ML yielded quantitative prediction vs HPLC		
Poplar-type	Turkey	*A.m. caucasica; A.m. anatolica; A.m. carnica*	3	Ethanol 70%	GC–MS	48 compounds; Populus & Salix markers	-	Bee speces origin	Antibacterial	Bee race behaviour differences influence propolis composition	2005	[41]
Poplar-type (P. nigra, P. tremula, P. × euramericana)	Serbia; Bosnia & Herzegovina; Bulgaria	*Apis mellifera*	59	Methanol (ultra-pure)	NMR; IR; UV-Vis	Phenolic glycerides; flavonoids	OPLS; O²PLS	Botanical (altitude-dependent origin)	-	High-altitude samples richer in phenolic glycerides; low-altitude dominated by flavonoids	2017	[42]
Poplar-type	Croatia	*Apis mellifera*	6	Ethanol 80%; UAE/maceration/MAE	HPLC; HPTLC; GC-MS; UV-Vis	Flavonoids; Phenolic acids	-	Geographical origin; QC	Antioxidant	HPTLC, HPLC and GC characterization and QC	2013	[43]
Poplar-type; Mixed-type	Poland; Romania; Turkey; Uruguay	*Apis mellifera*	8 (1 Poland; 1 Uruguay; 1 Turkey; 5 Romania)	Ethanol 70%	HPLC-DAD; TPC; TFC; DPPH/ABTS	CAPE; t-Cinnamic acid; Chrysin; Pinocembrin; Caffeic acid; Ferulic acid; Gallic acid; p-Coumaric acid; Apigenin; Hesperidin;	PCA HCA	Geographical origin	Antioxidant	European poplar type vs Turkish and Uruguayan	2022	[44]

						Pinocembrin						
Poplar-type tinctures	Hungary	*Apis mellifera*	252 (various regions)	Ethanol 80%	ICP-OES/ICP-MS (elemental transfer)	Essential/Toxic elements	Correlation analysis	Elemental QC; Geographical origin	-	Geographical authentication impracticable	2014	[45]
Romanian poplar-type	Romania	*Apis mellifera*	39	Ethanol 95%	TLC; Image Analysis	Phenolic band patterns	Fuzzy clustering; PCA	Geographical origin; Botanical origin	-	Meadow area vs Forest area	2011	[46]
Blue; Orange	Germany	*Apis mellifera*	64	Ethyl acetate	HPTLC; DART-MS	Caffeic acid; Naringenin; Apigenin; Quercetin; Kaempferol; Galangin; Chrysin; Ellagic acid	PCA; HCA; LDA	Subtype discrimination	-	Blue type vs Orange type discrimination	2014	[3]
Poplar-type	Italy	*Apis mellifera*	60	Ethanol	HR-NMR	Flavonoids; Phenolic acids	Factor Analysis; General Discriminant Analysis	Harvesting method	-	1H NMR (4.5–13 ppm) classified by harvesting method with 96.7% predictive capacity	2007	[47]
Poplar-type (Po Valley vs Apennines)	Italy (Po Valley; Ligurian–Piedmont Apennines)	*Apis mellifera*	12	Ethanol 70%	HPLC–Orbitrap-MS; NMR; HS-SPME GC–MS	Phenolic glycerides; Poplar resin markers	-	Geographical origin	Antioxidant	VOCs distinguish regions; phenolic glycerides dominate mountain samples	2020	[11]
Chinese poplar-type	China	*Apis mellifera*	12	Direct DHS (no extraction)	DHS-GC/MS; E-nose; GC-O	99 volatiles; odor-active compounds	PCA	Geographical origin	-	Geographical regions classified; key odorants	2013	[48]

										identified		
Chinese poplar-type	China	*Apis mellifera*	19	Methanol 80%; ultrasonication	HPLC-UV	Caffeic acid; Isoferulic acid; 3,4-Dimethoxycinnamic acid; Pinobanksin 5-methyl ester; Pinocembrin; Benzyl caffeate; Chrysin; Galangin	-	Routine QC	-	Robust simultaneous quantification	2009	[49]
Chinese poplar-type	China	*Apis mellifera*	37	Water; Ethanol 70%	nanoESI-MS; UPLC-MS/MS	Caffeic acid; p-Cinnamic acid; CAPE; Pinocembrin; Genistein; Ctric acid; Arctopicrn; Sinapinic acid; Benzoic acid; Gluconic acid; Quinic acid	PLS-DA; ANOVA; VIP Analysis; ML (RF, SVM, NN, LR, GB, SGD, Tree)	Climate-zone origin	-	Climate-zone and propolis-color authentication	2021	[50]
Chinese poplar-type	China	*Apis mellifera*	22	Ethanol (ultra-pure)	HPLC; enzyme assays	Catechol (new marker)	-	Adulteration detection	-	Catechol proposed as marker for poplar extract adulteration	2014	[7]
Poplar-type (Changbai Mountains, CBM)	China	*Apis mellifera*	21 + 49 + 104	Ethanol 95%; ultrasonication	HPLC-UV; HPLC-ESI/MS	p-Coumaric acid; Benzyl p-coumarate	-	Botanical origin	Antioxidant	New type defined by benzyl p-coumarate; source Populus davidiana/simonii	2019	[51]
Mixed-type, Multifloral	India	*Apis mellifera*	30 (Himachal Pradesh,	Ethanol 70%, ultrasonication	LC-ESI-QTOF-MS; RP-HPLC;	beta-Carotene; Galangin; CAPE	PCA; ANN	Geographical origin	Antioxidant	Regional charaterization of northern	2019	[69]

			Punjab, Haryana, Rajasthan)		TPC; TFC; DPPH/FRAP					Indian propolis		
Indian poplar-type	India	*Apis mellifera*	7 extracts + 14 supplements	Ethanol (ultra-pure)	UHPLC-QToF-MS; UHPLC-DAD; HPTLC	Pinocembrin; Chrysin; CAPE (14 phenolics validated)	-	Chemical composition; QC	Antioxidant	57 compounds identified; validated quantitative QC workflow	2020	[52]
Stingless-bee propolis (Geniotrigona thoracica)	Malaysia	*Stingless bee*	5	Methanol (ultra-pure)	HPTLC; FTIR	Flavonoids; Phenolics; Terpenoids	PCA; HCA	Geographical origin	-	Three clusters by location; FTIR + chemometrics classify effectively	2023	[53]
Non-Apis	Malaysia	*Stingless (Heterotrigona itama; Geniotrigona thoracica)*	2	Methanol 80%	TLC; Physicochemical parameters; DPPH	Steroids; Saponins; Coumarins	-	Species/geography differentiation	Antioxidant; Antidiabetic	Bee species authentication	2016	[5]
Blue; Orange; Green	Egypt	*Apis mellifera*	60	Ethanol 95%; ultrasonication	HPTLC-ESI-MS; UV; Palynology	3,4-Dimethoxycinnamic acid; Caffeic acid; Isoferulic acid; Rosmarinic acid; Quercetin	OPLS; PLS	Type discrimination; bioefficacy markers	Antidiabetic	HPTLC fingerprinting of 3 global propolis types; Palynological identification of 13 plant families	2022	[29]
Mixed-type	Ghana	*Apis mellifera*	3	Ethanol;	TPC;	Caffeic/quinic derivatives;	PCA; HCA; ANOVA	Source/solvent differentiation	Antioxidant;	Regional and solvent	2023	[54]

				Water; Ethanol 50%	TFC; DPPH; TLC	Quercetin; Naringenin; Hesperidin; Rosmarinic acid; Methyl cinnamate; Steroids; Triterpenoids			Antidiabetic	authentication		
Red propolis	Brazil	*Apis mellifera*	39	Ethanol 80%	UV-Vis; HPLC-DAD; LC-MS; DPPH	Flavonoids; Isoflavonoids; Polyprenylated Benzophenones	Correlation analysis; PCA; PLS-DA; OPLS-DA	Climate effect	Antioxidant; Antibacterial; Anti-trypanosomal	Climate-metabolite relationship	2019	[30]
Brazilian organic	Brazil	*Apis mellifera*	1	Ethanol	NMR; ABTS	Lignans (lariciresinol, secoisolariciresinol, pinoresinol, matairesinol)	-	Marker discovery	Antioxidant	Lignans as authenticity markers	2020	[55]
Brazilian brown	Brazil	*Apis mellifera*	7	Static headspace	SHS-GC-MS	Monoterpenes; Sesquiterpenes	PCA; HCA; heatmap	Botanical origin (resin source)	Antioxidant; Antimicrobial	Volatile profile matched Araucaria angustifolia resins; strong clustering	2021	[27]
Baccharis-type	Brazil (Southern;	*Apis mellifera*	59 (Southern Brazil, 3 agroecological regions; Autumn, Winter, Spring, Summer)	Ethanol 80%	NMR	Total flavonoids; Phenolic acids	PLS-DA; Random Forest	Seasonal/geographical classification	Antioxidant	Achieved seasonal discrimination stronger than regional	2015	[38]

Alecrim-type (Brazilian green)	Brazil	*Apis mellifera*	10 (different regions)	Volatile condensate; Hexane	TLC (simple visual method)	Allyl 3-prenylcinnamate	-	Baccharis-type confirmation	-	TLC-method for volatle marker of Brazilian alecrim propolis	2015	[37]
Stingless-bee; Green; Tubuna; Mandaçaia; Plebeia	Brazil	*Apis mellifera (scutellata); Scaptotrigona bipunctata; Melipona quadrifasciata; Plebeia remota*	4	Ethanol 77%	ATR-IR (FTIR); UPLC-qToF-MS/MS	Phenolic acids; Flavonoids; Prenylated cinnamates; Diterpenes	PCA; PLS-DA	Species/type authentication; QC	Antioxidant; Antimicrobial	Accurately classified four propolis classes; met Brazilian legislative criteria	2022	[56]
Poplar-type	Argentina	*Apis mellifera*	96	-	NAA	Trace minerals	PCA; LDA; kNN	Geographical origin	-	Elemental fingerprints for specific provenance	2011	[11]
Brown; Red; Yellow	Cuba	*Apis mellifera*	65	Methanol (ultra-pure)	HPLC-PDA; HPLC-MS; 1H NMR; 13C NMR	Polyisoprenylated benzophenones; Isoflavonoids; Pterocarpans	-	Type classification (color-based)	-	Three chemical types: brown=benzophenones; red=isoflavonoids; yellow=aliphatic	2003-2004	[57]
Poplar-type	Mexico (Sonoran desert)	*Apis mellifera*	12	Ethanol	1H-NMR; HPLC-UV-DAD	Pinocembrin; Pinobanksin; Chrysin; Galangin; Kaempferol;	PCA; HCA	Botanical origin	Anticancer	Botanical sources: Populus fremontii; Ambrosia	2019	[1]

Stingless bee (Melipona beecheii)	Mexico	*Melipona beecheii*	35	Ethanol 70%	UV-Vis (TPC; TFC)	Quercetin; p-Coumarc acid; Naringenin Total phenolics; Total flavonoids	PCA; HCA	Geographical origin	Antioxidant; Anti-inflammatory; Antimicrobial	ambrosioides; Bursera laxiflora Clustered samples by region and bioactivity	2023	[31]
Poplar-type	Belgium; Iran	*Apis mellifera*	2	Ethanol 70%	SDS-PAGE; MALDI-TOF MS; LC-MS	Poplar PR proteins; bee venom proteins	-	Botanical origin	-	Comprehensive proteome	2023	[58]
Mixed-type	Europe; Africa; Brazil; Asia; Solomon Islands	*Apis mellifera*	43	Ethanol	1H-NMR; HPLC	Caffeic acid; Cinnamic acid; Naringenin; Pinocembrin; Chrysin; Galangin	PCA	Geographical origin	Antioxidant	NMR outperformed single markers; Geographical authentication	2006	[59]
Poplar-type / Baccharis-type	Argentina; Brazil; China; Korea	*Apis mellifera*	12	Ethanol (ultra-pure)	UPLC; FT-ICR MS	Flavonoids; Phenols; Terpenoids; Fatty acids	PLS-DA	Geographical origin	-	Clear separation of propolis by country; marker families identified	2019	[28]
Commercial mixed-type propolis extracts (global)	Various (commercial products)	*Apis mellifera*	8	Ethanol and others	ATR-FTIR	FTIR bands: O–H; C–H; C=O; C–N; C–O	PCA; HCA	Geographical origin (commercial discrimination)	-	Samples effectively separated by origin	2025	[15]
16 high-grade types	Australia (QLD, NSW,	*Apis mellifera*	158	Ethanol 70%; ultrasonication	HPLC-UV; 1H NMR; DPPH assay	Phenolics; Flavonoids (chrysin;	PCA; PLS-DA	Geographical origin; QC	Antioxidant	Identified 16 high-grade types;	2022	[60]

	SA, WA, VIC, TAS)					pinocembrin; galangin; prenylated stilbenes; artepillin C)				several exceeded Brazilian green/red propolis in antioxidant capacity		
Poplar-type	China (17 provinces)	*Apis mellifera*	66 propolis + 8 poplar tree gum	Ethanol 95% (ultrasonication; 3× extraction); methanol re-extraction	RP-HPLC-UV	Flavonoids and phenolic acids: caffeic acid, ferulic acid, p-coumaric acid, isoferulic acid, cinnamic acids, pinobanksin, pinocembrin, 3-O-acetylpinobanksin, chrysin, galangin, CAPE	-	Adulteration (poplar tree gum); Quality grading; Routine QC	-	Validated RP-HPLC method for 20 phenolics; identified markers distinguishing propolis from poplar tree gum; established quantitative QC	2014	[63]
Poplar-type (Orange; Blue)	Serbia, Croatia, Slovenia (France, Brazil as outliers)	*Apis mellifera*	39 (23 Serbia; 9 Slovenia; 7 Croatia; plus 2 external)	Dichloromethane, ultrasonication	HPTLC; Image analysis	Flavonoids, phenolic acids; characteristic orange and blue TLC bands	PCA	Botanical origin (orange vs blue); Geographical origin (three countries)	-	HPTLC fingerprints + image analysis reliably distinguished orange vs blue European poplar-type; Serbia and Croatia; Slovenia; Brazilian sample	2016	[64]
Poplar-type (Orange; Blue)	Serbia	*Apis mellifera*	46	Ethanol 80%	UV–Vis; Cyclic voltammetry (CV); DPPH	Phenolic acids, flavonoids, caffeic acid derivatives,	PCA	Botanical origin (orange vs. blue poplar-type); Quality control	Antioxidant	Separated orange and blue propolis	2017	[65]

								parameters				
Brown propolis	Brazil	*Apis mellifera*	39 (South, East, West)	-	GC-MS	Resinic diterpenes (pimaric, isopimaric, dehydroabieti, abietic acids); aromatic acids (p-coumaric, vanillic, 2,5-dihydroxybenzoic acids); alcohols, hydrocarbons, aldehydes	PLS-DA	Geographical origin (regional differentiation within Paraná)	-	Strong regional separation among southern, eastern and western sectors	2024	[66]

*Propolis collection. Caffeic acid phenethyl ester (CAPE); ultra-high-performance liquid chromatography coupled to Orbitrap tandem mass spectrometry (UHPLC–LTQ/Orbitrap/MS/MS); inductively coupled plasma optical emission spectrometry (ICP-OEA); inductively coupled plasma mass spectrometry (ICP-MS); quality control (QC); genetic inverse least squares (GILS); thin-layer chromatography (TLC); bidirectional orthogonal partial least squares (O^{2}PLS); direct analysis in real time mass spectrometry (DART-MS); hierarchical cluster analysis (HCA); linear discriminant analysis (LDA); high-resolution nuclear magnetic resonance (HR-NMR); high-performance liquid chromatography with diode-array detection (HPLC-DAD); liquid chromatography–mass spectrometry (LC-MS); 2,2-Diphenyl-1-picrylhydrazyl (DPPH); partial least squares discriminant analysis (PLS-DA); orthogonal projections to latent structures discriminant analysis (OPLS-DA); neutron activation analysis (NAA); k-nearest neighbor (kNN); proton nuclear magnetic resonance spectroscopy (^{1}H-NMR); nanospray electrospray ionization mass spectrometry (nanoESI-MS); ultra-performance liquid chromatography tandem mass spectrometry (UPLC-MS/MS); variable importance in projection analysis (VIP Analysis); machine learning (ML); random forest (RF); support vector machine (SVM); neural network (NN); logistic regression (LR); gradient boosting (GB); stochastic gradient descent (SGD); decision tree algorithm (Tree); liquid chromatography–electrospray ionization quadrupole time-of-flight mass spectrometry (LC-ESI-QTOF-MS); reversed-phase high-performance liquid chromatography (RP-HPLC); total phenolic content (TPC); total flavonoid content (TFC); ferric reducing antioxidant power assay (FRAP); artificial neural network (ANN); orthogonal partial least squares/orthogonal partial least squares–discriminant snalysis (OPLS/OPLS-DA); partial least squares/partial least squares–discriminant analysis (PLS/PLS-DA); headspace solid-phase microextraction (HS-SPME); dynamic headspace gas chromatography–mass spectrometry (DHS-GC/MS); gas chromatography–olfactometry (GC-O); 2,2'-azino-bis(3-ethylbenzothiazoline-6-sulfonic acid) assay (ABTS); static headspace gas chromatography–mass spectrometry (SHS-GC-MS); attenuated total reflectance infrared spectroscopy (ATR-IR); proton nuclear magnetic resonance (^{1}H NMR); carbon-13 nuclear magnetic resonance (^{13}C NMR); sodium dodecyl sulfate polyacrylamide gel electrophoresis (SDS-PAGE); matrix-assisted laser desorption/ionization time-of-flight mass spectrometry (MALDI-TOF MS); ultra-performance liquid chromatography (UPLC); fourier transform ion cyclotron resonance mass spectrometry (FT-ICR MS).
